# A proof of concept study of ^18^F-FDG PET/CT patient-level radiomics identify refractory/relapsed diffuse large B-cell lymphoma

**DOI:** 10.1038/s41598-025-08223-8

**Published:** 2025-09-30

**Authors:** Caozhe Cui, Jianbo Cao, Yayuan Li, Boren Jia, Ning Ma, Xiaomeng Li, Meng Liang, Mingxia Hou, Yanan Zhang, Hongliang Wang, Zhifang Wu

**Affiliations:** 1 Special Medical Research Institute of Shanxi Medical University, Taiyuan, China; 2 Department of Nuclear Medicine, First Hospital of Shanxi Medical University, Taiyuan, China; 3 Collaborative Innovation Center for Molecular Imaging of Precision Medicine, Shanxi Medical University, Taiyuan, China

**Keywords:** ^18^F-FDG PET/CT, Diffuse large B cell lymphoma, Machine learning, Radiomics, Cancer imaging, Haematological cancer, Tumour heterogeneity

## Abstract

This study aimed to evaluate diffuse large B-cell lymphoma (DLBCL) patients who have refractory/relapsed disease and characterize the heterogeneity of DLBCL using patient-level radiomics analysis based on ^18^F-FDG PET/CT. A total of 132 patients diagnosed with DLBCL who underwent ^18^F-FDG PET/CT before receiving treatment were selected for the final study. Patient-level volumes of interests (VOI) were extracted from PET/CT images, and 328 radiomics features were extracted subsequently. 8 radiomics features were selected using the Least Absolute Shrinkage and Selection Operator (LASSO) algorithm to calculate the radiomics score (rad-score). Additionally, a total of 64 potential ML classifiers were generated based on 8 distinct supervised learning algorithms. The combined model that integrates rad-scores, clinical features and standard PET parameters demonstrates excellent performance; Specifically, ML models based on Naive Bayes have the greatest predicted values (AUC = 0.73). The patient-level radiomics features were subjected to unsupervised non-negative matrix factorization (NMF) clustering analysis to identify 3 radiomics subtypes. Cluster 1 exhibited a substantially higher prevalence of refractory/relapsed DLBCL compared to Clusters 2 and 3 (*P* < 0.05). Moreover, Cluster 1 showed a significantly higher frequency of advanced Ann Arbor stage, high international prognostic index, and bulk disease (all *P* < 0.05). In conclusion, Radiomics scores and radiomics subtypes derived from patient-level data offer significant predictive value and phenotypic information for patients with refractory/relapsed DLBCL.

**Supplementary Information:**

The online version contains supplementary material available at 10.1038/s41598-025-08223-8.

## Introduction

Diffuse large B-cell lymphoma (DLBCL) is the predominant aggressive subtype of non-Hodgkin lymphoma subtype in adults, characterized by widespread involvement and diagnostic challenges^1^. The conventional treatment for DLBCL is R-CHOP immunochemotherapy (which include the rituximab, cyclophosphamide, doxorubicin, vincristine, and prednisone), which aims to target cancer cells through various mechanisms, including direct cell killing and immune system regulation^2^. Although a significant number of patients, including those at advanced stages, achieve long-lasting survival through this approach, 10–15% patients encounter refractory disease and 20–30% relapse after an initial response^3,4^. Since rituximab is commonly administered during first-line chemotherapy, refractory or relapsed DLBCL patients may face worse outcomes than those in the pre-rituximab era^5^. Although the International Prognostic Index (IPI) can predict DLBCL patient outcomes, it falls short in accurately identifying individuals who are at risk of developing refractory or recurrent disease^6,7^. It is vital to promptly identify patients with refractory/relapsed DLBCL. And explore the alternative treatment options, such as salvage therapy, or clinical trials of novel drugs, ultimately enhancing their prognosis^4^.

DLBCL often involves multiple organs throughout the body, and these findings may be overlooked or misclassified on CT or MRI scans, especially when involvement is mild or atypical. ^18^F-FDG PET/CT is commonly employed for initial DLBCL staging and assessing first-line therapy response due to its effective to characterize tumor glucose metabolism and detect lymph node and extranodal involvement^8^. However, FDG-PET could produce false positive results due to inflammation. Conventional PET semi-quantitative parameters, such as maximum standardized uptake value (SUVmax) and total metabolic tumor volume (MTV), have been associated with the DLBCL prognosis^9,10^. Nonetheless, they are limited in fully describing subtle metabolic heterogeneity within targeted lesions. Tumor heterogeneity encompasses the variability in phenotypic characteristics of cancer cells, including cell structure, gene expression, metabolism, and metastasis capacity^11^. This complex phenomenon reflects not only genomic instability and epigenetic variability but also variations in prognostic outcomes among different tumor types^12,13^. Radiomics is an emerging era that involves extracting and analyzing vast amounts of quantitative data from medical images, such as CT or PET^14^. In the field of PET/CT radiomics in particular, the workflow necessitates detailed specification of key technical parameters to ensure methodological reproducibility, a critical requirement for both research validation and clinical translation^15^. These metrics derived from images effectively capture intra-tumoral and inter-tumoral heterogeneity associated with molecular and cellular characteristics^16,17^.

Despite awareness of the poor outcomes in patients with refractory/relapsed DLBCL, there is currently no efficient predictive tool used in clinical practice. At present, refractory/relapsed DLBCL is mainly explained through collaboration between nuclear medicine physicians and clinical physicians. Several studies^18,19^ focus solely on radiological characteristics of the lesion with the highest metabolic activity or largest volume, despite that a substantial proportion of DLBCL patients lack a distinct primary lesion, as the disease often disseminated throughout the body. Given the significant heterogeneity in patients and tumors, radiomics analyzing at the patient level may provide a more accurate representation of the unique variations in disease. This study aimed to evaluate the predictive value of baseline ^18^F-FDG PET/CT patient-level radiomics for refractory/relapsed DLBCL populations, and to investigates whether unsupervised learning-based radiomics subtypes could effectively characterize heterogeneity among DLBCL patients.

## Results

### Patient characteristics

The clinical characteristics of the patients are shown in Table 1, with the majority suffering from advanced diseases, elevated lactate dehydrogenase levels, and high IPI scores. Among the analyzed patients, 44 (33.33%) had refractory or relapsed DLBCL, while 88 (66.67%) did not. Statistically significant differences were found in Ann Arbor stage (*P* = 0.025), ECOG PS (*P* = 0.026), IPI (*P* = 0.009), and bulk disease (*P* < 0.001) between the refractory/relapsed DLBCL (RR) and non-refractory/relapsed DLBCL (NRR) groups. Table 2 provides a comprehensive list the PET characteristics for the patients. The RR and NRR groups exhibited significant differences in DmaxVox (*P* = 0.036), MTV (*P* < 0 0.001), and TLG (*P* = 0.006).

**Table 1:** Clinical characteristics of enrolled patients.

Characteristic	Whole population (*n* = 132)	RR group (*n* = 44)	NRR group (*n* = 88)	*p* value
Gender				0.806
Female	67 (50.8)	23 (52.3)	44 (50.0)	
Male	65 (49.2)	21 (47.7)	44 (50.0)	
BMI	23.1 ± 3.11	22.70 ± 3.04	23.30 ± 3.13	0.298
Age (year)				0.902
< 60	70 (53.0)	23 (52.3)	47 (53.4)	
≥ 60	62 (47.0)	21 (47.7)	41 (46.6)	
Ann Arbor stage				0.025
I-II	52 (39.4)	10 (22.7)	42 (47.7)	
III-IV	80 (60.6)	34 (77.3)	46 (52.3)	
Extranodal involvement				0.448
0–1	81 (61.4)	25 (56.8)	56 (63.6)	
≥ 2	51 (38.6)	19 (43.2)	32 (36.4)	
LDH				0.802
Normal	53 (40.2)	17 (38.6)	36 (40.9)	
Elevated	79 (59.8)	27 (61.4)	52 (59.1)	
ECOG PS				0.026
0–1	97 (73.5)	27 (61.4)	70 (79.5)	
≥ 2	35 (26.5)	17 (38.6)	18 (20.5)	
IPI				0.009
≤ 2	72 (54.5)	17 (38.6)	55 (62.5)	
> 2	60 (45.5)	27 (61.4)	33 (37.5)	
B symptoms				0.612
No	82 (62.1)	26 (59.1)	56 (63.6)	
Yes	50 (37.9)	18 (40.9)	32 (36.4)	
Bulk disease				0.001
No	88 (66.7)	21 (47.7)	67 (76.1)	
Yes	44 (33.3)	23 (52.3)	21 (23.9)	

Data expressed as count with percentage in parentheses, mean ± SD, or median with IQR in parentheses.

LDH: lactate dehydrogenase; ECOG PS: Eastern Cooperative Oncology Group perfor mance status; IPI: International Prognostic Index.

**Table 2:** Conventional PET parameters of enrolled patients.

PET Parameters	RR group (*n* = 44)	NRR group (*n* = 88)	*p* value
SUVmax	18.77 (8.73,25.2)	17.08 (8.45,25.88)	0.856
SUVmean	6.93 (4.32,9.97)	6.37 (3.87,9.12)	0.431
SUVstd	2.47 (0.95,4.23)	2.09 (1.12,4.39)	0.900
COV	0.35 (0.23,0.46)	0.36 (0.25,0.47)	0.460
Dmax	27.65 (19.58,38.50)	24.30 (13.50,38.50)	0.117
DmaxVox	37.00 (26.90,54.15)	27.20 (18.75,47.37)	0.036
MTV	280.02 (90.56,271.00)	88.50 (33.50,271.00)	0.001
TLG	1299.50 (476.00,4915.25)	487.50 (111.25,2686.75)	0.006

Data expressed as median with IQR in parentheses.

SUVmax: maximum standardized uptake value; TMTV: total metabolic tumour volume; TLG: total lesion glycolysis.

### Radiomics score development

**Fig. 1:**
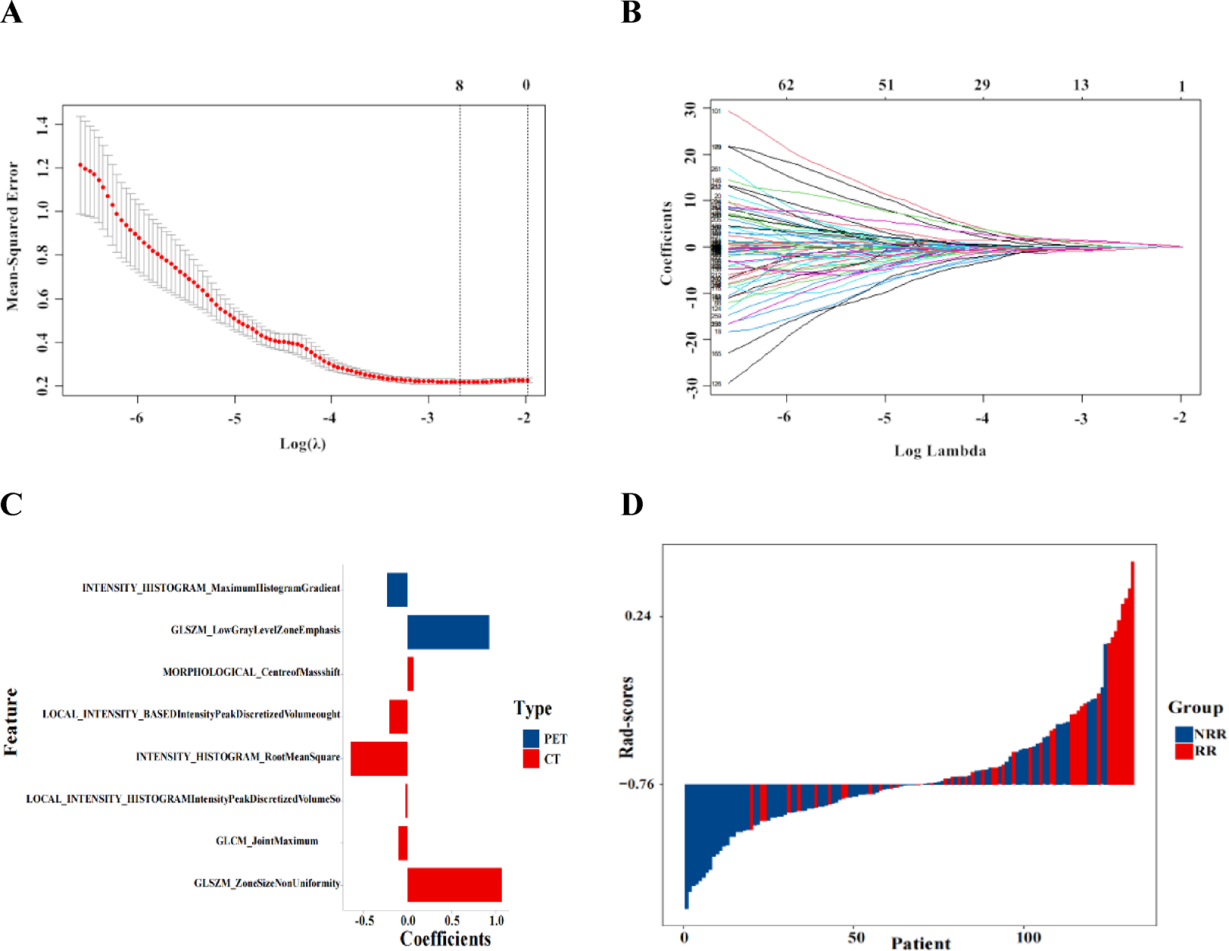
Construction of radiomics scores. Radiomics features selection using the Least Absolute Shrinkage and Selection Operator (LASSO) regression with 10-fold cross-validation. (**A**) The optimal Lambda value was identified by the minimum mean-squared error (MSE) and by the minimum MSE within one standard error. (**B**) LASSO coefficient profiles of the radiomics features. (**C**) Specific coefficients corresponding to each radiomics feature obtained by selection. (**D**) Waterfall plot obtained from radiomics scores of all patients.

By using the LASSO regression model, we refined 8 original features with an optimal λ value of 0.0686 (Fig. 1A and B). The rad-score was derived based on the corresponding coefficients of 8 radiomics features selected (Fig. 1C). For a detailed breakdown of radiomics features and their coefficients, see Table S1 in the Supplementary Materials. The median value was 0.76, and the RR group exhibited a significant higher rad-scores (*P* < 0.001) compare to the NRR group. Rad-scores were shown in a bar chart, and their distribution is displayed in Fig. 1D.

### Machine learning model performance

MTV had a strong correlation with TLG (*r* = 0.94, *P* < 0.001). Given the established prognostic superiority of MTV (Figure S1), this parameter was prioritized for integration into the multivariate predictive modeling framework^9,19^. The clinical model was constructed based on significant clinical features (Ann Arbor stage, ECOG PS, IPI, Bulk disease). Conventional PET features (MTV and DmaxVox) were used to construct PET prediction models. The above models were used to predict refractory/relapsed DLBCL. To prevent overfitting of the composite model, the combinations of variables were further screened. Ultimately, the clinical-PET model was built using IPI, bulk disease, MTV, and DmaxVox with rad-scores being added to produce the combined model. For a detailed description of the prediction models included in this study, see Table S2 in the Supplementary Materials.

**Fig. 2:**
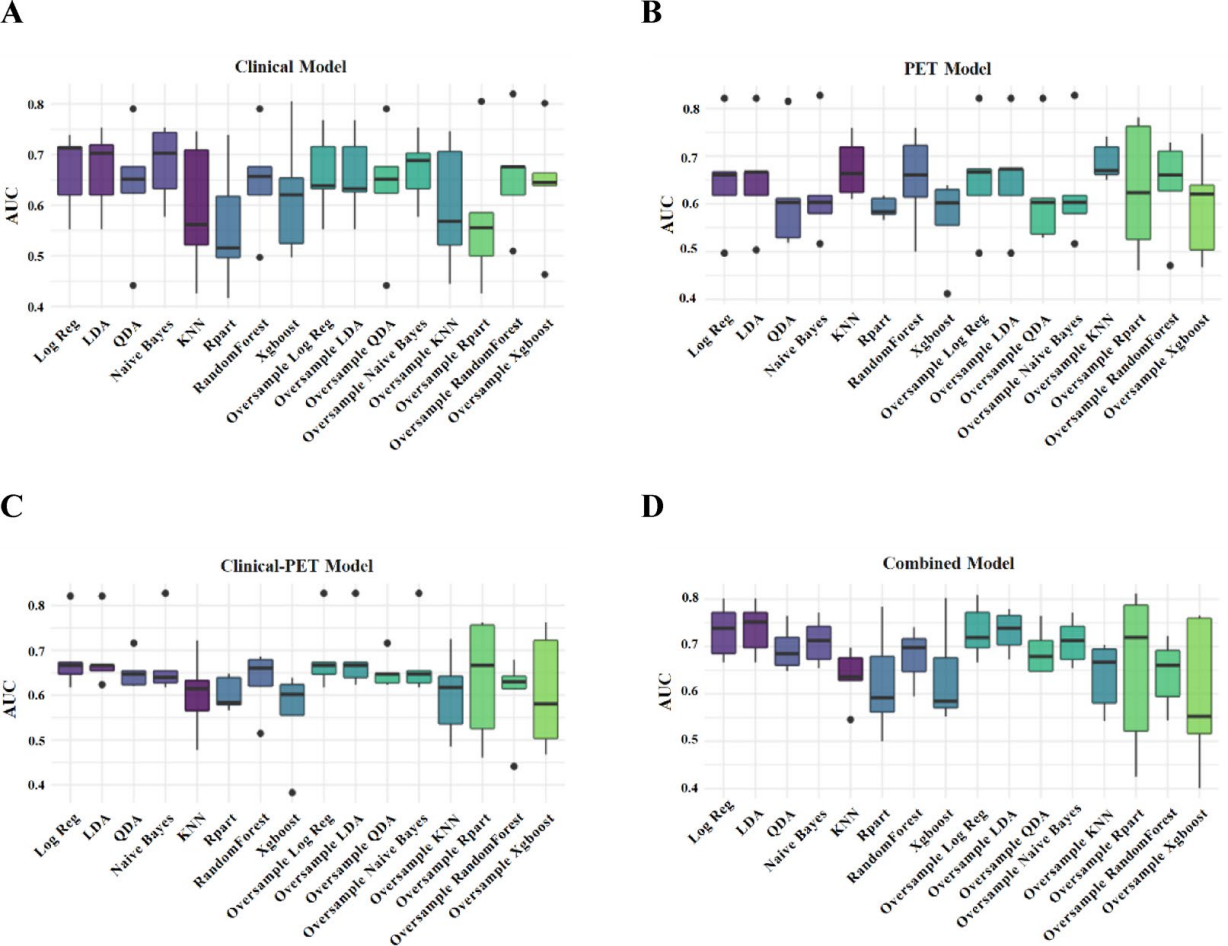
The efficiency of ML. Box plots, showing performance of each model and eight machine learning models (with and without oversampling) for predicting outcomes in test set. Horizontal line in box indicated median; ends of box indicate IQR; ends of whiskers indicate interdecile range; and points beyond whiskers indicate outliers. (**A**) AUC values of clinical models. (**B**) AUC values of PET models. (**C**) AUC values of clinical-PET models. (**D**) AUC values of Combined models.

Figure 2 presents the performance evaluation of the 64 ML models for predicting refractory/relapsed DLBCL using 8 algorithms (with or without oversampling). For a detailed of the predictors for all models, see Table S3 in the Supplementary Materials. The combined model based on Naive Bayesian Algorithm produced the highest AUC (0.73) and the highest accuracy (0.69) among all ML models with the oversampling applied. In the clinical model, Naive Bayes algorithm had the highest AUC (0.68), and Rpart had the highest accuracy (0.69). In the PET model, the KNN algorithm with oversampling had the highest AUC (0.7). Additionally, the three ML algorithms (Log Reg, LDA, and KNN) produced the highest accuracy (0.67). The clinical-PET Model, yielded the best AUC with Log Reg and LDA algorithms, and both accuracy improved with oversampling.

### Radiomics subtype characterization

**Fig. 3:**
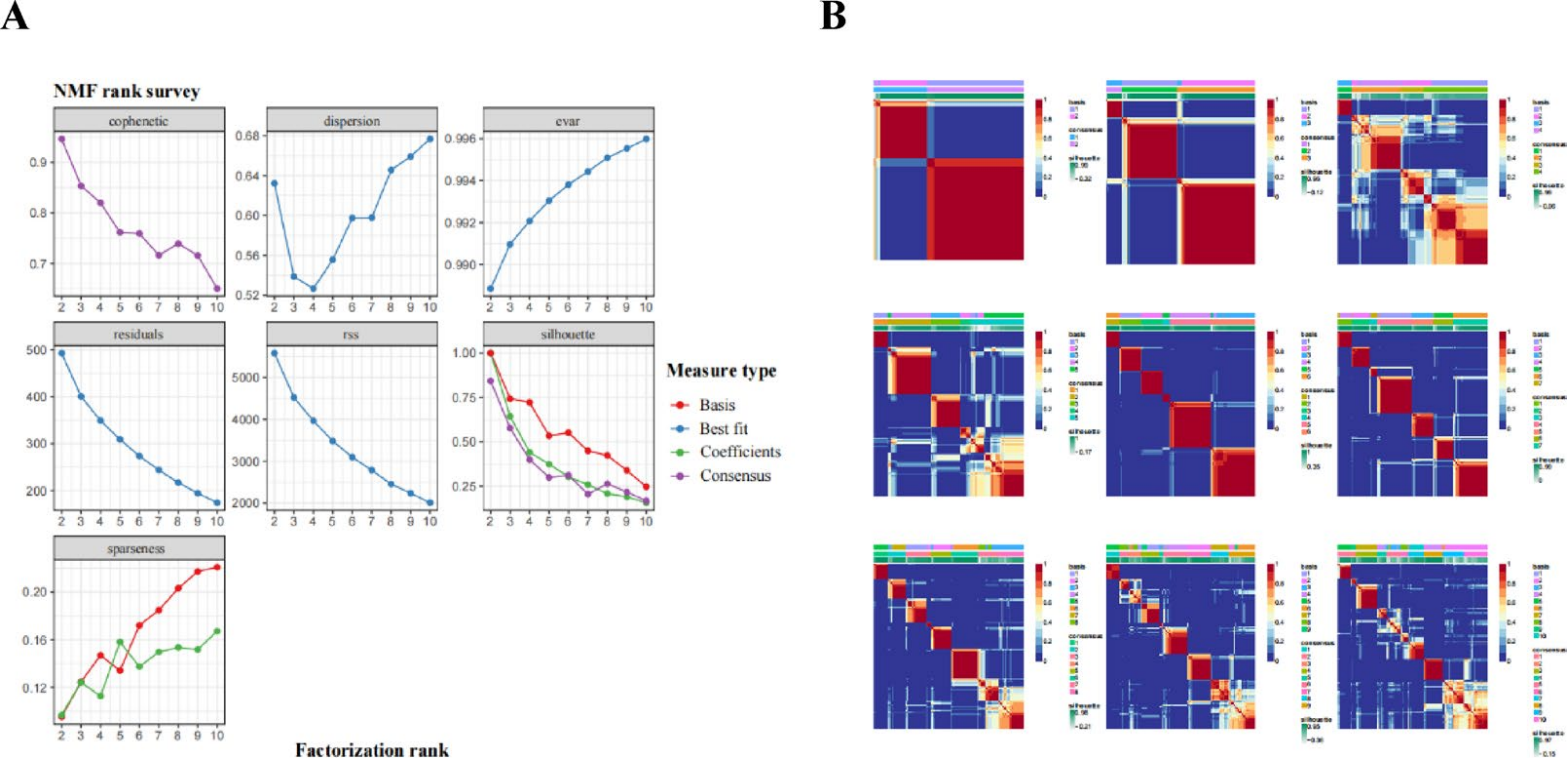
The results of NMF. Identification of DLBCL radiological subgroups based on differential radiomics features. (**A**) Consensus matrix heatmap with K ranging from 2 to 10. (**B**) Relationship between cophenetic, dispersion, evar, residuals, rss, silhouette, and sparseness coefficients with respect to the number of clusters.

**Fig. 4:**
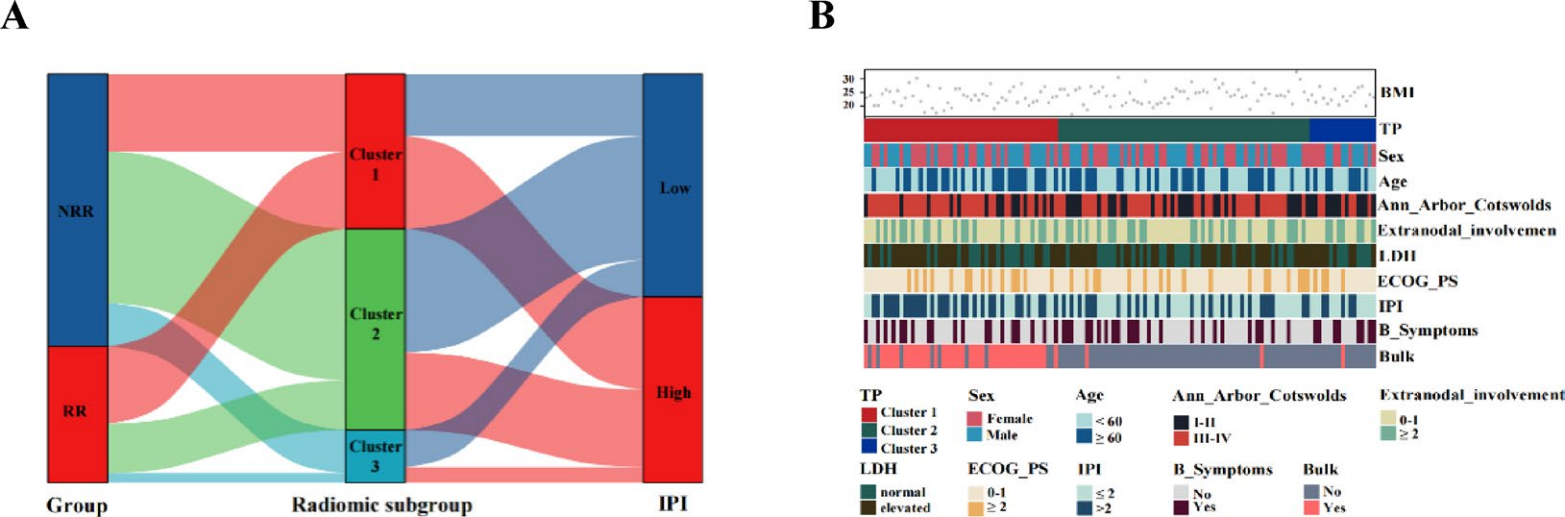
Comparison between radiomics subtypes. Differences in radiomics subgroups. (**A**) The Sangi diagram shows the population grouped by RR/NRR, radiomics subgroups, IPI, and other characteristics, showing the patterns of different groups. (**B**) Heatmap with clinical characteristics of the 3 clusters.

We examined the statistical association between patients in the RR and NRR groups. A total of 29 differential radiomics features were selected after conducting the difference analysis across groups. Using cophenetic, dispersion and silhouette curves (Fig. 3), we identified 3 robust clusters (radiomics subtypes), with 50, 65 and 17 patients in clusters 1, 2 and 3, respectively. The incidence of refractory/relapsed DLBCL was significantly higher in Cluster 1 (25 of 50 [50%]) compare to Cluster 2 (16 of 65 [24.6%]) and Cluster 3 (3 of 17 [17.6%]) groups (*p* = 0.006) (Fig. 4A).

Clinical characteristics grouped into clusters are summarized in Fig. 4B. For a detailed of Clinical characteristics, see Table S4 in the Supplementary Materials. In cluster 1, 82% patients (41out of 50) had a higher frequency of Bulk disease, which exhibited a significant higher occurrence compared with clusters 2(2 of 65 [3.1%]) and 3(1 of 17 [5.9%]) (*P* < 0.001). Similarly, the cluster1 had the highest frequency of High IPI (30 of 50 [60%]) compare to cluster 2 (25 out of 65[38.5%]) and cluster 3 was 5 of 17 (29.4%) (*P* = 0.026). There was significant difference in Ann Arbor stage between the 3 clusters (*P* = 0.025), with a higher frequency of high Ann Arbor stage in cluster 1 (36 of 50 [72%]) than in cluster 3(6 of 17 [35.3%]). There was no significant difference in other clinical features between the 3 clusters (all *P* > 0.05).

Conventional PET features grouped into clusters are summarized in Table 3. There were no significant differences between the 3 clusters, only for SUVstd and COV (all *P* > 0.05). Cluster 1 had the highest median fraction of SUVmax (20.36 [IQR = 12.91–26.94], *P* = 0.011) and SUVmean (7.70 [IQR = 5.78–10.21], *P* = 0.011). There was significant difference in Dmax among the three clusters(*P* = 0.008), with a higher median fraction of Dmax in cluster 1 than in cluster 3. Similarly, the median fraction of DmaxVox was highest in cluster 1 (41.35 [IQR = 28.07–56.72], *P* = 0.001). Cluster 1 had a higher median fraction of MTV (520.63 [IQR = 348.75–1174.71], *P* < 0.001) and TLG (4373 [IQR = 2298–7737.25], *P* < 0.001) compared to clusters 2 and 3.

**Table 3:** Conventional PET parameters by different Cluster.

PET Parameters	Cluster 1 (*n* = 50)	Cluster 2 (*n* = 65)	Cluster 3 (*n* = 17)	*p* value
SUVmax	20.36 (12.91,26.94)	13.53 (6.76,23.24)	16.09 (9.51,23.05)	0.011
SUVmean	7.70 (5.78,10.21)	5.20 (2.81,8.04)	6.22 (4.69,10.68)	0.008
SUVstd	2.72 (1.44,4.27)	1.81 (0.80,4.75)	2.39 (1.22,4.38)	0.532
COV	0.33 (0.24,0.43)	0.39 (0.24,0.52)	0.34 (0.32,0.43)	0.241
Dmax	33.00 (20.03,45.38)	25.90 (13.50,38.40)	16.50 (9.00,23.00)	0.008
DmaxVox	41.35 (28.07,56.72)	27.60 (17.10,44.80)	22.40 (15.20,31.70)	0.001
MTV	520.63 (348.75,1174.71)	56.00 (27.00,103.50)	82.00 (35.00,131.00)	< 0.001
TLG	4373.00 (2298.00,7737.25)	262.00 (85.00,642.00)	355.00 (114.00,540.00)	< 0.001

Data expressed as median with IQR in parentheses.

## Discussion

The study highlights the significance of patient-level radiomics analysis using baseline ^18^F-FDG PET/CT in predicting refractory/relapsed DLBCL. This finding supports the hypothesis that whole-body PET/CT imaging may offer additional metabolic information for tumor heterogeneity evaluation and potentially guide risk stratification. The study was based on a retrospective cohort of 132 patients, and the developed combination of rad score, clinical data, and PET features generally performed better than clinical and/or PET models alone. It is suggested that rad-scores may provide additional value for predicting refractory/relapsed DLBCL by conventional factors such as IPI and MTV. Among all ML models evaluated, the combined model based on the Naive Bayes algorithm had the highest efficiency. Unsupervised learning was employed to analyze radiomics data from DLBCL patients in an unbiased manner, resulting in the effective categorization of patients in 3 radiomics subtypes using NMF. Multiple clinical and conventional PET parameters were significantly different between different radiomics subtypes. Higher frequency of refractory/relapsed DLBCL was found in cluster 1.

The heterogeneity observed in DLBCL patients is likely attributed to biological diversity, and both clinical and molecular heterogeneity may have an impact on the prognosis of patients. Identifying predictive biomarkers of refractory/relapsed DLBCL is crucial for treatment selection and clinical decision support. Currently, most research has focused on biomarkers identified in biological samples, such as peripheral blood and tumor tissue^20–22^. Rushton et al.^23^ sequenced circulating tumor DNA from 135 refractory/relapsed DLBCL and found that TP53 and KMT2D were mutated in the majority of these patients, indicating a role in first-line treatment for drug resistance. Metabolic reprogramming such as glycolysis, plays a crucial role in the formation of tumors. The heterogeneity in tumor glucose uptake is potentially visualizable by ^18^F-FDG PET/CT^13^. In our study, we computed two parameters to characterize the tumor heterogeneity: rad-score and COV.

Figure 1 demonstrates that the rad-scores, comprised of multiple radiomics features, were significantly different between groups and more accurately represented heterogeneity. This finding aligns with a recent study identifying radiomics signature and IPI as independent risk factors for 2-year PFS and OS of DLBCL^24^. However, as shown in Table 2, there was no significant difference in COV among the groups. Previous studies have demonstrated that COV is capable of measuring tumor heterogeneity and has the potential to serve as a prognostic risk factor for primary mediastinal large B-cell lymphoma patients^35^. In our research, the region of interest was based on patient-level analysis rather than focusing on a single lesion, this approach may account for the inadequacy of a basic measurement such as COV in capturing the diverse metabolic characteristics of the patients. As DLBCL often involves multiple lymph nodes and extranodular masses and exhibiting a high heterogeneity of intertumoral and intratumoral ^18^F-FDG uptake within the same individual. Based on these findings, we suggest that utilizing patient-level rad-scores derived from PET/CT scans may provide a more accurate representation of individual variations.

DLBCL, a systemic malignancy without typical primary tumor, is usually diagnosed through biopsy of a single lesion in routine clinical practice. Thus, exploring tumor heterogeneity over the entire tumor volume may be more relevant to patient outcomes than a single lesion in DLBCL^25^. However, measuring total MTV in DLBCL remains challenging compared to other tumors like lung cancer. Furthermore, analysis and interpretation of ^18^F-FDG PET images are usually performed by experienced physicians. While a standardized whole-body PET imaging segmentation method has yet to be established for DLBCL. This may have some impact on the robustness of PET features. Barrington et al.^34^ compared several segmentation methods on PET images of DLBCL and determined that automatically estimating the total MTV by SUV 4.0 to be a feasible approach. Eertink et al.^26^ examined 6 different semi-automatic segmentation methods for radiomics analysis, discovering that the classification performance of radiomics features remained consistent, regardless the variations in specific radiomics feature values obtained through the different segmentation methods. In our study, we employed segmentation with SUV 4.0, complemented by manual modifications made by experienced physicians, to achieve the whole lesion segmentation. This approach boasts a high segmentation rate, with over half of the patients requiring only simple clicking to remove uptake in the brain, urinary tract, and heart. However, the automated process was not entirely reliable in some cases, particularly when dealing with neighboring lesions or areas with high physiological uptake in the gastrointestinal tract, which necessitated time-consuming user interaction.

ML models based on radiomics have increasingly been incorporated into cancer imaging research. Our study demonstrates that the rad-scores calculated by LASSO was correlated with refractory/relapsed DLBCL. The patient-level model based on radiomics score has a high value in predicting refractory/relapsed DLBCL. Similarly, recent research also supports the potential of these radiomics features in predicting progression after 2 years for DLBCL patients. However, variations in both the extracted features and the quantity of characteristics makes it difficult to directly compare different studies^27,28^. Another study exploring the analysis of multiple single lesions in vivo has revealed that a random forest model may effectively identify primary treatment failure in refractory DLBCL patients^7^. Our study had a majority of patients without refractory/relapsed disease, resulting in an imbalance in the results, which we corrected by creating synthetic samples. Similar to the Jiang et al.^29^ study, we compared the combined model based on multiple ML algorithms with other models, such as clinical model and PET model. Figure 2 and Table S3 showed that the combined model based on Naive Bayes algorithm had the highest AUC, supporting the feasibility of combining radiomics features with clinical predictors. A study was conducted to predict the relapsed or refractory disease status of 251 Hodgkin lymphoma patients with radiomics features extracted from baseline PET scans, and the results showed that the model constructed with 5 radiomics features was superior to MTV, TLG^30^. In contrast, to prevent radiomics features from overwhelming clinical indicators, our study combined multiple radiomics features to construct rad-score, which was then combined with other factors to improve the clinical applicability of the model. The metrics in the combined model include multimodal data for both clinical and images. Considering that the complex biological processes of DLBCL may occur at various scales, integrating clinical and imaging factors could offer a more comprehensive understanding of disease characteristics and enhance predictive efficacy and accuracy.

Supervised methods primarily identify candidate biomarkers of known phenotypes, while unsupervised methods seek to uncover new subgroups and potentially detect relevant variables. Most studies on tumor subtypes focus on genomics, with only a few exploring radiomics^31^. The identification of radiomics subtypes refers to the process where patients or diseases are stratified into different groups based on quantitative imaging features, which could have implications for diagnosis, prognosis, and personalized treatment. Perez-Johnston et al.^32^ identified 4 radiomics clusters in I-stage lung adenocarcinoma CT scans and investigated clinical-pathological characteristics and genomic associations between the different clusters. As shown in Fig. 3,, we divided DLBCL patients into 3 radiomics subtypes using NMF based on differential radiomics features of baseline PET/CT, which suggested different efficacy profiles and tumor heterogeneity. These 3 radiomics subtypes exhibited significant differences in multiple clinical and PET factors. Half of the DLBCL patients in cluster 1 had refractory/relapsed disease, implying this subtype may have a poor prognosis; in contrast, the frequency of refractory/relapsed DLBCL in cluster 3 was below average. Among clinical factors, cluster 1 had more advanced stage patients, higher IPI scores, and a higher incidence of bulk disease; In terms of PET, cluster 1 also has a high median of MTV, TLG and DmaxVox. These factors have previously been associated with refractory/relapsed DLBCL in other studies and our results. Interestingly, cluster 2 had the lowest median of SUV conventional parameters (SUVmax, SUVmean, SUVstd). Given the heterogeneity of DLBCL, our findings suggest that radiomics clusters could be utilized for DLBCL patient classification, potentially enabling more personalized treatment approaches in the future.

This study has a few limitations. This study is a retrospective study with a small sample size, patient selection bias cannot be eliminated. It should be noted note that our results were not verified by an independent cohort of participants, although we did evaluate their reliability through internal validation using cross-validation. Second, radiomics were extracted from the total target area at the patient level, but total MTV measurement methods were inconsistent across different working groups. The possible impact of this technical issue on the generality of the conclusions. Furthermore, current molecular genetic studies have proposed DLBCL subtypes with poor prognosis, such as activated B-cell subtypes or MYC oncogene rearrangements. However, only a subset of patients in our cohort underwent subtype analysis. Further investigation is needed to determine if imaging biomarkers derived from ^18^F-FDG PET are associated with different DLBCL subtypes. Finally, this study did not consider the possibility of identifying feature selection frequency during cross validation, which may have an impact on model performance.

In this study, we developed machine learning models based on radiomics scores that were able to distinguish refractory/relapsed DLBCL patients by baseline ^18^F-FDG PET/CT; Additionally, radiological phenotypic analysis based on NMF is used to obtain relevant categories of DLBCL patients with significantly different risk of refractory/relapsed development. In summary, our findings demonstrate the potential of patient-level radiomics analysis as a method for evaluating refractory/relapsed DLBCL from both supervised and unsupervised ML perspectives, potentially contributing to more personalized treatment approaches in the future.

## Methods

### Patients

**Fig. 5:**
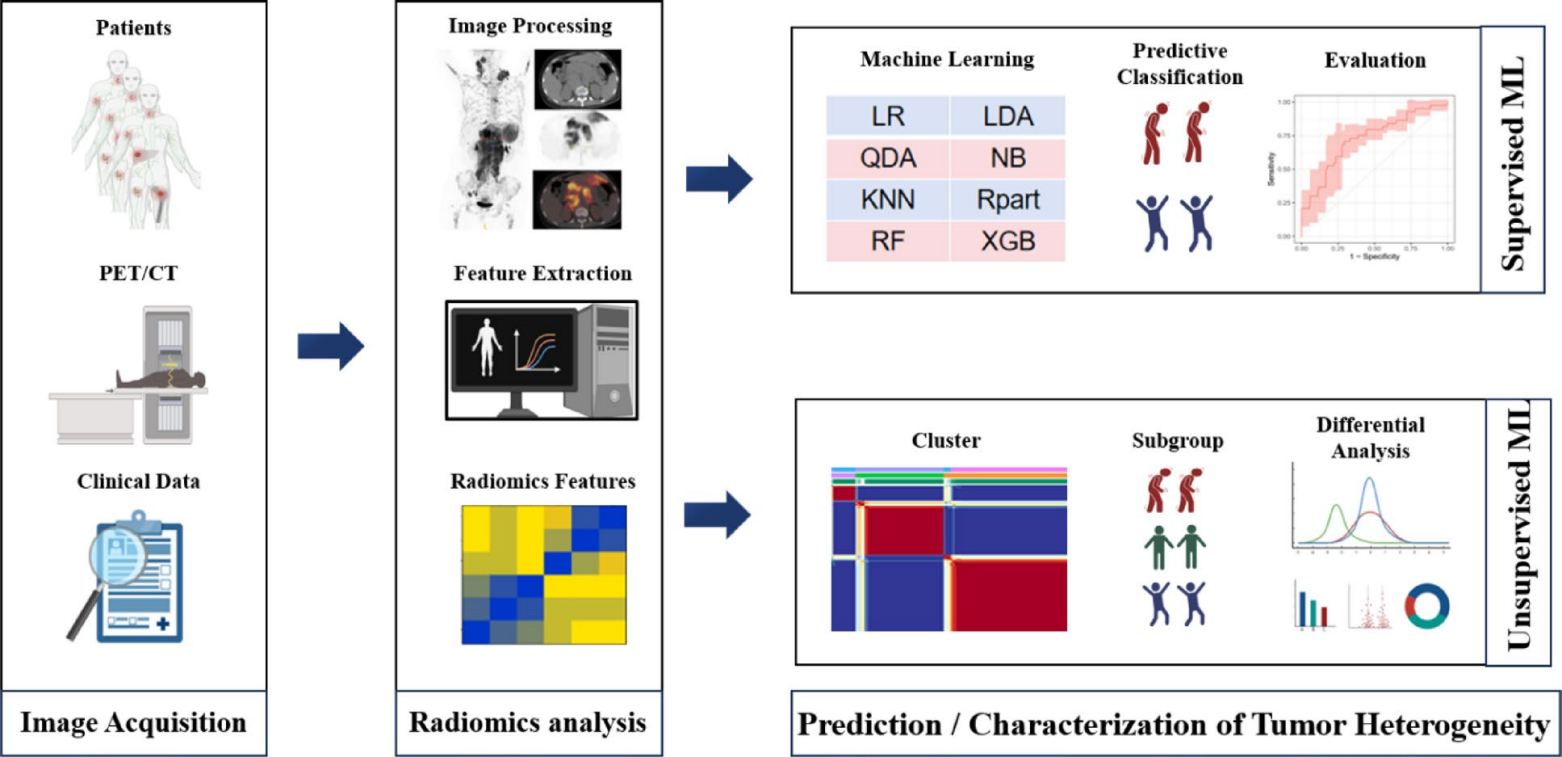
Schematic study flowchart.

This study has been approved by the Medical Ethics Committee of the First Hospital of Shanxi Medical University and obtained informed consent from patients (No. KYLL-2024-280). The study strictly followed the Declaration of Helsinki. We retrospectively enrolled patients diagnosed with DLBCL who underwent ^18^F-FDG PET/CT examination at the First Hospital of Shanxi Medical University between August 2017 and December 2022 by querying the hospital’s medical record system. The study was carried out in accordance with the principles outlined in the Declaration of Helsinki through ethical clearance and obtained informed consent from all patients. The eligibility criteria mandated that patients fulfilled the following conditions: (1) having a pathologically confirmed diagnosis of DLBCL; (2) underwent clinical treatment with R-CHOP or a similar regimen; (3) exhibiting ^18^F-FDG-avid lesions on PET scans; and (4) over 18 years of age. Exclusion criteria: (1) Prior therapeutic interventions preceding PET/CT imaging acquisition; (2) Incomplete imaging datasets or lesions below 10 cc volumetric threshold for reliable quantification; (3) Insufficient clinical documentation for comprehensive analysis. Patients were ineligible if they had prior treatment before PET/CT scans, had incomplete or low-quality images that were challenging to analyze, or had no valid clinical data. Of the 143 patients included in the study, 4 patients without complete clinical data were excluded; To ensure stability and interpretability of imaging features, 7 patients were excluded (including 1 patient had diffuse liver involvement, 2 patients had diffuse inflammatory hypermetabolism postoperatively, and 4 had lesions smaller than 10 cc). This yielded a total of 132 patients who were analyzed. Figure 5 presents a schematic study flowchart.

All patients underwent a comprehensive medical history assessment, laboratory tests, and an ^18^F-FDG PET/CT scan. Additional information that was collected included: age, Ann Arbor stage, Eastern Cooperative Oncology Group (ECOG) performance status, lactate dehydrogenase (LDH) levels, and extranodal involvement. Tumor masses exceeding a diameter of 7.5 cm were categorized as bulky disease. Refractory DLBCL was defined as progressive or stable disease with first-line treatment of chemotherapy greater than 4 cycles or later treatment of 2 cycles^3,4^. Relapsed disease reflects the appearance of new lesions after attainment of complete response^3,4^.

### PET/CT scanning protocol

All patients were scanned using a GE Healthcare Discovery MI PET/CT scanner (GE Healthcare, USA) after fasting for a minimum of 6 h and maintaining their blood glucose levels below 200 mg/dl. The ^18^F-FDG was administered intravenously at a dose of 2.96–4.44 MBq (0.08–0.12 mCi) per kilogram of body weight. Following a waiting period of 50–60 min in a quiet environment, a CT scan was performed from the top of the skull to the middle of the femur, with a tube voltage of 120 kV, a tube current of 60–230 mA, and 3.75 mm slice thickness. PET acquisition was then subsequently performed over the same range, with 5–7 bed positions acquired for 3 min/bed. The matrix size of PET/CT images is 256 × 256, with voxel sizes of 0.98 × 0.98 × 3.3 mm (CT) and 5.5 × 5.5 × 3.3 mm. The PET data were automatically attenuated using the CT data to obtain PET reconstruction and PET/CT fusion images, which then were stored on the workstation in DICOM format.

### Image segmentation and feature extraction

The ^18^F-FDG PET/CT images were evaluated by two nuclear medicine specialists (10 and 20 years’ experience receptively) who were blinded to patient outcomes. To minimize the inter-observer variability, all images were analyzed by the same observer with LIFEX 7.3 software^33^, and confirmed by an experienced senior nuclear medicine specialist. PET image volume of interest (VOI) segmentation was performed using the semi-automated segmentation module in the LIFEX. The lesion delineation protocol comprised three sequential optimization phases: (1) Primary segmentation was performed using semi-automated algorithm with dual-threshold parameters (SUVmax ≥ 4.0; minimum volume 3 mL) to generate initial volumetric contours; (2) Systematic refinement involved manually excluding physiological ^18^F-FDG biodistribution patterns (myocardial, cerebral, renal parenchymal, and urinary bladder activity) alongside non-malignant tracer retention sites; (3) Complementary augmentation addressed potential under-segmentation through expert-guided inclusion of subthreshold metabolic foci (SUVmax 2.5–4.0) and small volume metabolic lesions (The volume size 1–3 ml) exhibiting morphospatial continuity with primary lesions^34^. All VOIs were merged to form a consolidated patient-level VOI for PET. The patient-level VOIs of PET were verified with fused PET/CT images and transferred to corresponding CT slices.

For extranodal involvement, liver, lung, and bone marrow involvement were considered only if there was a focal uptake. Homogeneous bone marrow uptake was excluded from the tumor volume, while splenic involvement was defined as focal uptake or diffuse uptake higher than 150% of the liver background.

The standardized uptake value (SUV) was used as a scaling factor for the voxel values, based on the net injection tracer dose per kilogram of body weight. Five conventional patient-level SUV features were retrieved without any resampling: SUVmax, SUVmean, SUVstd, MTV and total lesion glycolysis (TLG). In addition, dissemination characteristics (Dmax and DmaxVox) and heterogeneity parameters (COV) were calculated. Dmax refers to the euclidean distance between the centre locations of two lesions that are farthest apart; While DmaxVox represents the distance between the two lesions farthest away using the outermost voxels. For the patient had only one lesion, the Dmax and DmaxVox values were both recorded as 0 cm. COV, an intuitive and straightforward parameter, was defined as the ratio of SUVstd to SUVmean within an automatically segmented tumor volume to estimate heterogeneity of ^18^F-FDG uptake^35^.

Prior to identifying radiomics features, we preprocessed both PET and CT images, including resampling all PET images to a voxel size of 1 × 1 × 1 mm³ using bilinear interpolation and discretizing PET images with a fixed bin count of 64^36^. CT images were subjected to the same resampling procedure employing a fixed bin count of 256. Based on previous research^24^ radiomics feature extraction was performed on patient level VOI. The patient level VOI quantification was performed through LIFEx software’s integrated whole-volume preservation algorithm (“save all in one” function), enabling comprehensive radiomic characterization across all metabolically active tumor subregions. A total of 328 radiomics features were extracted, including grayscale histogram parameters, grayscale co-occurrence matrix (GLCM, Haralick), neighborhood grayscale difference matrix (NGLDM, Amadasum), grayscale run length matrix (GLRLM, Xu), and grayscale zone length matrix (GLZLM, Thibault). (comprehensive descriptions of these textures were available at http://www.lifexsoft.org). These radiomics features comply with the feature definition described by the Imaging Biomarker Standardization Initiative (IBSI)^37^.

### Radiomics analysis and model construction

Min-Max normalization [x* = (*x* − *min*)/(*max* − *min*), *max* is the maximum value of the sample data, and *min* is the minimum value of the sample data] was applied to all radiomics features. All radiomics features were scaled to within [0,1] to reduce the influence of different dimensions. To minimize overfitting, highly correlated features (with correlation coefficients greater than 0.9) were removed. Then, the LASSO regression algorithm was employed to further select the remaining features. LASSO adds L1 regularization terms to the least squares algorithm to reduce overfitting. The Lambda parameters were optimized using a 10-fold cross-validation technique. The most optimal Lambda value was determined for which the cross-validation error was within one standard or error of the minimum. To identify relevant features that may differentiate refractory/relapsed DLBCL, coefficients of irrelevant features were set to zero. The selected radiomics features were used to compute rad-score for each patient.

ML algorithms may handle data with complex non-linear relationships to clinical outcomes. We developed prediction models for clinical features, conventional PET features, rad-scores, and various combinations of features using 8 different machine learning (ML) methods (Table S5): Logistic Regression (Log Reg), Linear Discriminant Analysis (LDA), Quadratic Discriminant Analysis (QDA), Naive Bayes, K-Nearest Neighbor (KNN), Rpart, RandomForest, and Xgboost. The stratified 5-fold cross-validation method was utilized to assess the generalizability of the model. We trained on 80% of the data and validated on a 20% unseen subset in each cross-validation fold. Up until each fold was utilized as a test set, this procedure was repeated. Oversampling of patients with refractory/relapsed DLBCL was performed to correct for imbalance in the classification of patients with refractory/relapsed DLBCL and non-refractory/relapsed DLBCL^28^. We created 44 positive samples and 0 negative samples using SMOTE, resulting in a 1:1 ratio of positive to negative samples in the total population. We performed all pre-processing and optimization steps on each training fold to prevent test data from contaminating the trained model. To evaluate model performance, we calculated the area under the ROC curve (AUC), accuracy, sensitivity, and specificity for each model using ROC curve analysis.

### Radiomics subtypes analysis

To investigate whether patient-level radiomics features reflect heterogeneity among patients with DLBCL, we analyzed radiomics features between groups and considered those with a *p* value of < 0.05 to be differential. We then used non-negative matrix decomposition (NMF) in unsupervised clustering to identify the radiomics matrix of DLBCL patients, an approach that allows identification of the appropriate number of clusters based on differences in tumor texture features. Radiomics subtypes have been constructed using NMF to differentiate patients with different risk levels based on similarities and differences in tumor texture features. To identify this optimal number of clusters, a range of K values is typically explored. Various evaluation metrics, such as cophenetic or dispersion, can be calculated for each K value to assess the quality and separation of the resulting clusters. The range is often determined based on prior knowledge of the dataset or through empirical observation. We set k to range from 2 to 10 and divided DLBCL samples into different clusters. Finally, we examined the correlation between clinical and PET features of each radiomics subtype obtained by clustering.

### Statistical analysis

All statistical analyses were conducted using R (version 4.0) and SPSS software (version 25.0, IBM). We assessed the difference in related clinical and PET characteristics between the refractory/relapsed and non-refractory/relapsed DLBCL groups using either the Chi-square or Mann-Whitney U tests, depending on appropriateness. We used the Chi-square or Kruskal-Wallis H test to compare characteristics across different radiomics subtypes. All statistical comparisons were two-sided, and *p* < 0.05 was considered statistically significant.

## Electronic supplementary material

Below is the link to the electronic supplementary material.

Supplementary Material 1

## Data Availability

The datasets used and/or analyzed during the current study available from the corresponding author on reasonable request.

## Code availability

The custom code and machine learning algorithms we use are open source, and readers can access the custom code through GitHub (DOI: 10.5281/zenodo.15087401).
