# Supplementary material for: A proof of concept study of 18F-FDG PET/CT patient-level radiomics identify refractory/relapsed diffuse large B-cell lymphoma
Source: Sci Rep. 2025 Sep 30;15:33914. doi: 10.1038/s41598-025-08223-8 (PMC12484957; doi:10.1038/s41598-025-08223-8)
Supplement: Supplementary file 1 — Supplementary Material 1 [file 41598_2025_8223_MOESM1_ESM.docx]

**
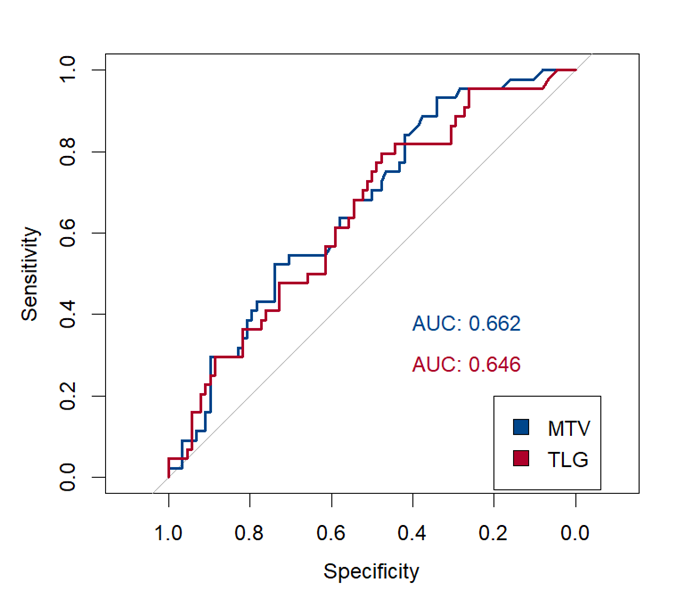
**

**Figure S1 ROC curves for predicting R/R DLBCL using MTV and TLG**

**Table S1 Selected 8 radiomics features and coefficients**

| Radiomic Features | Coefficients |
| --- | --- |
| PET INTENSITY_HISTOGRAM_MaximumHistogramGradient | -0.22892275 |
| PET GLSZM_LowGrayLevelZoneEmphasis | 0.92336278 |
| CT MORPHOLOGICAL CentreofMassshift | 0.07017484 |
| CT LOCAL_INTENSITY_BASEDIntensityPeakDiscretizedVolumeought. | -0.20462196 |
| CT INTENSITY_HISTOGRAM RootMeanSquare. | -0.64143927 |
| CT LOCAL_INTENSITY_ISTOGRAMIntensityPeakDiscretizedVolumeSought | -0.01993085 |
| CT GLCM_Joint Maximum. | -0.10151023 |
| CT GLSZM_ZoneSizeNonUniformity | 1.06293024 |

**Table S2 Description of prediction models included in this study**

| Models | Included Variables |
| --- | --- |
| Clinical Model | Ann Arbor stage, ECOG PS, IPI, Bulk disease |
| PET Model | MTV, DmaxVox |
| Clinical-PET Model | IPI, Bulk disease, MTV, DmaxVox |
| Combined Model | IPI, Bulk disease, MTV, DmaxVox, Rad-score |

**Table S3 The performance evaluation of the 64 machine learning models for predicting refractory/relapsed DLBCL using 8 algorithms**

| Models | Clinical  Model | | PET  Model | | Clinical-PET Model | | Combined  Model | |
| --- | --- | --- | --- | --- | --- | --- | --- | --- |
|  | AUC | Accuracy | AUC | Accuracy | AUC | Accuracy | AUC | Accuracy |
| Log Reg | 0.67 | 0.63 | 0.66 | 0.67 | 0.69 | 0.65 | 0.71 | 0.66 |
| LDA | 0.67 | 0.64 | 0.67 | 0.67 | 0.69 | 0.67 | 0.72 | 0.68 |
| QDA | 0.64 | 0.65 | 0.65 | 0.65 | 0.65 | 0.66 | 0.68 | 0.65 |
| Naïve Bayes | 0.68 | 0.65 | 0.65 | 0.67 | 0.67 | 0.67 | 0.73 | 0.67 |
| KNN | 0.59 | 0.38 | 0.67 | 0.61 | 0.60 | 0.60 | 0.63 | 0.65 |
| Rpart | 0.56 | 0.69 | 0.65 | 0.63 | 0.60 | 0.57 | 0.59 | 0.64 |
| RandomForest | 0.65 | 0.66 | 0.66 | 0.66 | 0.63 | 0.60 | 0.66 | 0.65 |
| Xgboost | 0.62 | 0.62 | 0.57 | 0.59 | 0.56 | 0.53 | 0.64 | 0.63 |
| Oversample Log Reg | 0.66 | 0.62 | 0.66 | 0.61 | 0.69 | 0.70 | 0.71 | 0.65 |
| Oversample LDA | 0.66 | 0.62 | 0.67 | 0.60 | 0.69 | 0.68 | 0.73 | 0.65 |
| Oversample QDA | 0.64 | 0.58 | 0.65 | 0.66 | 0.65 | 0.66 | 0.68 | 0.65 |
| Oversample Naïve Bayes | 0.67 | 0.65 | 0.65 | 0.64 | 0.67 | 0.68 | 0.73 | 0.69 |
| Oversample KNN | 0.60 | 0.38 | 0.70 | 0.62 | 0.60 | 0.58 | 0.64 | 0.58 |
| Oversample Rpart | 0.57 | 0.55 | 0.53 | 0.51 | 0.63 | 0.63 | 0.59 | 0.57 |
| Oversample RandomForest | 0.66 | 0.61 | 0.64 | 0.61 | 0.60 | 0.57 | 0.62 | 0.59 |
| Oversample Xgboost | 0.64 | 0.59 | 0.63 | 0.64 | 0.61 | 0.57 | 0.57 | 0.58 |

**Table S4 Clinical Characteristics by Different Cluster**

| Clinical  Characteristic | Cluster 1  (n = 50) | Cluster 2  (n = 65) | Cluster 3  (n = 17) | P-value |
| --- | --- | --- | --- | --- |
| Gender |  |  |  | 0.978 |
| Female | 25(50.0) | 33(50.8) | 9(52.9) |  |
| Male | 25(50.0) | 32(49.2) | 8(47.1) |  |
| BMI | 22.69 ± 3.06 | 23.46 ± 3.31 | 22.98 ± 2.38 | 0.589 |
| Age (year) |  |  |  | 0.644 |
| < 60 | 24(48.0) | 36(55.4) | 10(58.8) |  |
| ≥ 60 | 26(52.0) | 29(44.6) | 7(41.2) |  |
| Ann Arbor stage |  |  |  | 0.025 |
| I-II | 14(28.0) | 27(41.5) | 11(54.7) |  |
| III-IV | 36(72.0) | 38(58.5) | 6(35.3) |  |
| Extranodal involvement |  |  |  | 0.973 |
| 0-1 | 31(62.0) | 40(61.5) | 10(58.8) |  |
| ≥ 2 | 19(38.0) | 25(38.5) | 7(41.2) |  |
| LDH |  |  |  | 0.813 |
| Normal | 20(40.0) | 25(38.5) | 8(47.1) |  |
| Elevated | 30(60.0) | 40(61.5) | 9(52.9) |  |
| ECOG PS |  |  |  | 0.933 |
| 0-1 | 36(72.0) | 48(73.8) | 13(76.5) |  |
| ≥ 2 | 14(28.0) | 17(26.2) | 4(23.5) |  |
| IPI |  |  |  | 0.026 |
| ≤ 2 | 20(40.0) | 40(61.5) | 12(70.6) |  |
| > 2 | 30(60.0) | 25(38.5) | 5(29.4) |  |
| B Symptoms |  |  |  | 0.677 |
| No | 31(62.0) | 42(64.6) | 9(52.9) |  |
| Yes | 19(38.0) | 23(35.4) | 8(47.1) |  |
| Bulk disease |  |  |  | <0.001 |
| No | 9(18.0) | 63(96.9) | 16(94.1) |  |
| Yes | 41(82.0) | 2(3.1) | 1(5.9) |  |

**Table S5 Selection and Parameter Application of Machine Learning Algorithms**

| Machine learning algorithms | Advantage Scenarios | Key Tuning Parameters |
| --- | --- | --- |
| Log Reg | Linear classification, small data | penalty, C, L1_ratio, max_iter |
| LDA | Strong class separability, multi-dim | method, nu, tol |
| QDA | Strong class separability, multi-dim | Gamma, Lambda |
| Naive Bayes | High-dimensional sparse data | Laplace, adjust |
| KNN | Local pattern recognition, no training | Number of neighbors (k), distance, kernel |
| Rpart | High interpretability | Minsplit, cp |
| RandomForest | High-dim data, noise resistance | min.node.size, mtry |
| Xgboost | Large-scale data, competition scenarios | Eta, max_depth, subsample, min_child_weight, nrounds |
